# Psychosocial correlates of perceived stress among undergraduate medical students in Nigeria

**DOI:** 10.5116/ijme.59c6.3075

**Published:** 2017-10-26

**Authors:** Bawo O. James, Ibironke F. Thomas, Joyce O. Omoaregba, Esther O. Okogbenin, Kingsley M. Okonoda, Abdu W. Ibrahim, Auwal S. Salihu, Yewande O. Oshodi, Andrew Orovwigho, Paul C. Odinka, George O. Eze, Godwin C. Onyebueke, Benjamin E. Aweh

**Affiliations:** 1 Department of Clinical Services, Federal Neuropsychiatric Hospital, Benin City, Nigeria; 2 Synapse Services Centre for Psychological Medicine, Lagos, Nigeria; 3 Department of Psychiatry, College of Medicine, Ambrose Alli University, Ekpoma, Edo State, Nigeria; 4 Department of Psychiatry, University of Jos, Plateau State, Nigeria; 5 Department of Mental Health, College of Medical Sciences, University of Maiduguri, Borno State, Nigeria; 6 Department of Psychiatry, Bayero University, Kano, Nigeria; 7 Department of Psychiatry, College of Medicine, University of Lagos, Lagos State, Nigeria; 8 Department of Clinical Services, Federal Neuropsychiatric Hospital, Enugu, Enugu State, Nigeria; 9 Department of Psychological Medicine, College of Medicine, University of Nigeria, Nsukka, Enugu State, Nigeria; 10 University of Sierra Leone Teaching Hospital, Freetown, Sierra Leone; 11 Enugu State University of Science and Technology, Parklane, Nigeria; 12 Irrua Specialist Teaching Hospital, Edo State, Nigeria

**Keywords:** Medical students, stress, Nigeria, psychosocial correlates

## Abstract

**Objectives:**

To assess the prevalence and factors associated with perceived stress among medical students.

**Methods:**

A cross-sectional study of students (n=623) selected across eight medical schools in Nigeria. A structured questionnaire obtained socio-demographic characteristics, alcohol use (Alcohol Use Disorders Identification Test), other psychoactive drug use (Drug Abuse Screening Test), anxiety/depression symptoms (Hospital Anxiety Depression Scale) and stress (Perceived Medical School Stress Scale). We performed bivariate analysis using the chi-squared test, t-test and one-way ANOVA, with multiple regression analysis for multivariate testing in analysing the data.

**Results:**

Most students reported experiencing medical school stress. Female participants were more likely to perceive medical school as competitive (t_(621)_=1.17, p=0.003), less likely to see medical school as a threat (t_(621)_=-2.70, p=0.01) or worry about finances (t_(621)_=-4.80, p=0.001). Nearly a quarter; 21.3% (n=133) and 28.6% (n=178) reported depression and anxiety symptoms respectively. Approximately 4.2% (n=26) were dependent on alcohol, while 14.1% (n=88) had ‘low-risk use’ for other psychoactive substances. In the multiple regression model, lack of finance (B=2.881, p=0.001), weak adherence to religious faith (B=2.376, p=0.001), anxiety symptoms (B=-2.231, p=0.002), problematic alcohol use (B=5.196, p=0.001) and choice of study influenced by parents (B=-3.105, p=0.001) were predictors of greater perceived stress.

**Conclusions:**

Medical students in Nigeria report high levels of stress. Incorporating stress reduction strategies in the medical curriculum, and the input of students in providing feedback regarding the methods and styles of undergraduate medical education is required.

## Introduction

Research on stress or perceived stress among undergraduate students in general and medical students is growing.^1^^-^^6^ The available body of evidence suggests that certain factors either singly or in combination contribute to the development or perpetuation of stress in undergraduate programs.^5^^,^^7^^,^^8^ Factors include those unique to the individual, that either protect, predispose, or perpetuate stress, or factors considered ‘environmental’. ‘Personal’ factors include personality predisposition, coping style, psychological and physical health, psychoactive substance use and religious adherence.^5^ ‘Environmental’ factors, on the other hand, may comprise the curriculum, living and study conditions, as well as financing.^9^^,^^10^

Medical students consistently report higher levels of stress or perceived stress when compared to other students or the general population.^4^^,^^11^ Differences in cultural and geographical setting, study design, year of study of the medical student sample, and assessment tools may explain variation in the prevalence rates.

Modifications of the transactional model of stress underlie study designs in earlier reports.^5^ Vitaliano classed students into ‘resistors’, whose stress levels did not increase over their training period, ‘persistors’ whose scores were high at baseline and remained so over their course of study. The third; ‘adaptors’ whose high scores at baseline reduced over the course of study and ‘maladaptors’ whose low scores at baseline worsened over the course of study.^7^ They also identified personality, anger expression and coping as distinguishing factors among the groups.^5^^,^^8^^,^^12^^,^^13^ A recent report confirms via robust structural equation models, that personal resources by way of resilience, self-efficacy, and optimism impact on perceived stress. These affect how individuals cope with the demands of study, tension from interpersonal interactions and worries from expectations and consequences of studying.^5^ Perceived stress also mediates how emotional distress links with symptoms of anxiety and depression.^6^^,^^14^^,^^15^

A sizable body of evidence exists in the literature on perceived stress and its psychosocial correlates. However most studies conducted in Europe, the Middle East, and North America cannot be generalised to sub-Saharan Africa. In Nigeria, the period of undergraduate medical education has been reported as a particularly stressful,^16^ with students reporting high levels of stress.^17^ This study aimed to ascertain the magnitude of perceived stress among medical students and compare findings with reference samples from other countries. In addition, it aimed to explore the pattern of perceived stress and identify if any, gender differences in perceived stress. Certain factors (financial status, motivation to study the course, religious adherence, psychoactive substance use and emotional health) identified from published literature to either improve or worsen stress were tested to determine the association with perceived stress in this cohort.

## Methods

### Study design and sample size

This was a cross-sectional descriptive study of undergraduate medical students in their clinical years at accredited medical schools (public and privately owned) in Nigeria. Medical education in Nigeria comprises three years of pre-clinical studies followed by three years of clinical coursework, after 12 years of basic primary and secondary education. There are 31 medical schools in the country. Twenty-seven are fully accredited and four partially accredited.^18^

Thirty-one medical schools are spread across six geo-political zones. We selected eight accredited medical schools using probability sampling: University of Lagos (South-West), the University of Nigeria and Enugu State University (South-East), Bayero University (North-West), University of Maiduguri (North-East), University of Jos (North Central) and Ambrose Alli University and Igbinedion University (South-South). We recruited 635 students from the eight selected medical schools.

At each university, the Ethics Committee (IUO & AAU: Ethics Committee FNPH, Benin-City; BUK: Bayero University Ethics Review Committee; UNILAG: University of Lagos Ethics Review Committee; UNIMAID: University of Maiduguri Ethics Review Board; UNIJOS: Jos University Ethics Review Committee; ESUT & UNN: Enugu State University Ethics Committee) reviewed and approved the study protocol. We assured each participant of the confidentiality of their responses. We collected no identifying information to ensure anonymity. We assured students that no adverse consequence would result from their decision not to participate in the study as participation was voluntary. They were also informed that findings would be used for research purposes only and disseminated in a scientific conference or a peer review journal. 

At each selected medical school, we invited medical students in their clinical years (400-600 levels) to participate in the study. We randomly selected a class from the 400 to 600 levels. In a selected class, all students were invited after at a randomly selected lecture, to participate. At each site, the researcher ensured that the lecture selected was not the one he/she was delivering to prevent bias. The site researcher explained the nature and purpose of the study and obtained written consent. We distributed the self-administered questionnaire and retrieved completed ones 30 minutes after. We made efforts to ensure adequate spacing during class due to the sensitive nature of some questions to allow unbiased responses. We excluded students who did not attend the randomly selected lecture. Ninety-four students from all the sites did not participate in the randomly selected lecture. These students were similar in age and gender when compared with participants. The study took place between November 2014 and August 2015.

### Instruments

#### Socio-demographic Questionnaire

We designed this questionnaire to capture variables such as age, gender, marital status, religious adherence, as well as a source of funding for the medical school.

#### Perceived Medical School Stress Scale

Stress perception among students was measured using the adapted version, 15 of the Perceived Medical School Stress (PMSS) questionnaire.^7^^,^^12^ It comprises 13 items that present response options on a five-point Likert scale, ranging in the original version and the results of Holm et al. (2010) from "strongly disagree: 0" to "strongly agree: 4". Total scores range between 0 and 52, with higher scores indicating greater perceived stress. In a recent study, the Cronbach’s alpha coefficient for the PMSS was 0.81.^19^ In this study; the PMSS had good face validity and internal consistency with a Cronbach’s alpha of 0.74.

#### Alcohol Use Disorder Identification Test

The Alcohol Use Disorders Identification Test (AUDIT) is a simple ten-question test developed by the World Health Organisation to determine harmful alcohol use. Responses are scored between 0 and 4, with total scores ranging between 0 and 40. Higher scores indicate a greater risk of alcohol-related problems. The test was designed to be used internationally and was validated in a study using patients from six countries.^20^ Questions 1–3 deal with alcohol consumption, 4–6 relate to alcohol dependence and 7–10 consider alcohol-related problems. A validation study in Nigeria recommended a cut-off of 5 for hazardous alcohol use, with cut-offs of 7 and 9 for harmful use and alcohol dependence respectively.^21^  The AUDIT demonstrated good sensitivity; 100% and specificity; 92.1% in a recent study from Nigeria. ^22^

#### Drug Abuse Screening Test

The Drug Abuse Screening Test (DAST) is a 10-item self-administered tool that provides a quick assessment of drug use problems. Initial psychometric assessment involved a cohort of 256 clients with substance use problems.^23^ The DAST10 is as reliable as the longer DAST-20. The DAST-10 has been validated in the varied setting; substance-abuse patients,^24^ primary care,^25^ in the workplace,^26^ and adapted for use with adolescents.^27^ Suggested scoring for the DAST-10: “0 No problem”, “1-2 Low level”, “3-5 Moderate level”, “6-8 Substantial level”, “9-10 Severe level”.^23^

#### Hospital Anxiety and Depression Scale

Hospital Anxiety and Depression Scale (HADS),^28^screens for anxiety and depression symptoms. The HADS is a 14-item scale; seven items each relate to anxiety and depression. Each item response derives a score from 0-3, with total scores for each subscale ranging between 0 and 21. Caseness on the HADS has been determined with at least a score of 8 out of 21. Abiodun also obtained an optimum cut-off point of 8 for the HAD anxiety and depression sub-scales in a validity study conducted in Nigeria.^29^

### Data analysis

We coded data into an electronic spreadsheet (SPSS version 20). We summarized the data using descriptive statistics and presented them in tables. We tested associations between the dependent variable (perceived stress total score) and categorical dependent variables using the Student’s t-test/one-way ANOVA). For analysis, age was dichotomised into: ‘≥24 years’ and ‘<24 years’; type of accommodation was dichotomised into: ‘on-campus hostel’ and ‘others’. We regrouped adherence to faith into ‘strong’ (very strong, strong), ‘fair’ (fair), and ‘poor’ (very poor, poor). Similarly, AUDIT scores were dichotomised into two groups: ‘No problem’ and ‘Problem drinking’. Scores on the DAST-10 were dichotomised into: ‘No problem’ and ‘Problematic use’. The scores on the anxiety subscale and the depression subscale of the HADS respectively were dichotomised into: ‘absent’ and ‘present’. Significant associations on bivariate testing between perceived stress and psychosocial correlates were entered into a multiple regression model to identify predictors of perceived medical school stress. We set the level of significance a priori at p<0.05.

## Results

### Socio-demographic characteristics of medical students

We analysed questionnaires from 635 students. Thirteen incompletely filled questionnaires were and excluded from the analysis. Therefore we analysed a total of 623 questionnaires. Of the 623 medical students who participated in this study, 359 (57.6%) were male. The mean age was 23.91 (±3.67) years. Most participants were single (n=509, 81.7%) and Christian (n=459, 73.7%). Most did not have a previous higher degree (n=556, 89.2%), and the majority reported that they obtained funding for their education from their parents or a close relative (n=545, 87.5%). Participants described the ease of funding as “easy but could be better” (51.2%), while 5.6% found it “difficult” to fund their education. Over half (n=356, 57.1%) lived in hostels on the university campus. The ‘desire to help others’ was a common factor that had motivated most students (63.3%) to choose to study medicine. See Tables 1 and 2.

### Clinical characteristics of medical students

A majority did not report problematic drinking when assessed on the AUDIT (n=570, 91.5%) however, twenty-six students (4.2%) were dependent on alcohol. Similarly, a majority reported no problems on the DAST-10 regarding use of other psychoactive substances, although 14.1% of them had ‘low risk’ problems with the use of psychoactive substances. One hundred and seventy-eight students (28.6%) were screen positive for anxiety, while 21.3% were screen positive for depressive symptoms as rated by the HADS (see Table 3).

**Table 1:** Socio-demographic characteristics of participants

Variable		Frequency	%
Age class			
	<24 years	310	49.8
	>24 years	313	50.2
Mean age (SD)		23.91 (3.67)	
Gender			
	Female	264	42.4
	Male	359	57.6
Marital status			
	Single	553	88.8
	Co-habiting	9	1.4
	Married	61	9.8
Religion			
	Christianity	459	73.7
	Islam	161	25.8
	None	3	0.5
Adherence to faith			
	Very strong	154	24.7
	Strong	239	38.4
	Fair	178	28.6
	Poor	42	6.7
	Very poor	10	1.6
Previous higher degree			
	Yes	67	10.8
	No	556	89.2

### Pattern of perceived medical students stress

Table 4 shows the scores obtained on the 13 items of the PMSS. The mean scores ± SD for an item were highest on items 7; ‘medical school more competitive than I expected’ (2.40±1.16), 4; ‘medical training controls my life’ (2.27±1.28), and 12; ‘concern about personal finances’ (2.18±1.28). Female students were significantly more likely to report that medical school was competitive; (2.56±1.14 vs. 2.28±1.17, t(621) =2.95, p=0.003) and less likely to see medical school as more of a threat than a challenge (1.30±1.15 vs. 1.56±1.25, t(621)=-2.70, p=0.01) and be less concerned about personal finances (1.90±1.29 vs. 2.39±1.24, t(621) = -4.80, p<0.001).

**Table 2:** Psychosocial characteristics of participants

Variable		Frequency	%
Source of funds			
	Self-funded	44	7.1
	Parents/family	545	87.5
	Scholarship	34	5.4
Ease of funding			
	Very easy	123	19.7
	Easy, but could be better	319	51.2
	Difficult	146	23.4
	Very difficult	35	5.7
Accommodation type			
	On-campus residence	356	57.1
	Off-campus residence	205	32.9
	Family residence	62	10.0
What influenced decision?			
	Parents’ choice	56	9.0
	Role model	94	15.1
	Financial stability	77	12.3
	Desire to help others	396	63.6

### Correlates of medical school-related stress

Students who hold a previous higher degree reported significantly less stress compared to those who held none (22.36±6.63 vs. 24.37±7.99, t_(__621)_=-1.98, p=0.048). Those who found it ‘easy’ to fund their education also had significantly lower stress levels (22.02±8.77 vs. 24.68±7.55, t_(__621)_=-3.37, p=0.001). Students who choose to study medicine out of a desire to help others had significantly less stress (F_(__3/621)_ =10.40, p<0.001). Participants who rated their adherence to their faith as poor were significantly more likely to report greater stress (F_(__2/621)_ =39.44, p<0.001).

**Table 3:** Pattern of psychoactive substance use and psychiatric morbidity

Variable		Frequency	%
Alcohol use (AUDIT)			
	No problem	570	91.5
	Hazardous	16	2.6
	Harmful use	11	1.7
	Dependence	26	4.2
Other psychoactive substance use (DAST-10)			
	No problem	493	79.1
	Low risk	88	14.1
	Moderate risk	33	5.3
	Substantial risk	4	0.6
	Severe risk	5	0.8
Anxiety (HADS-A)			
	Absent	445	71.4
	Present	178	28.6
Depression (HADS-D)			
	Absent	490	78.7
	Present	133	21.3

Students with ‘problem drinking’ reported significantly more stress (29.74±8.37 vs. 23.63±7.63, t_(__621)_=-5.53, p<0.001). Presence of anxiety (26.54±7.95 vs. 23.20±7.65, t_(__621)_=-4.87, p<0.001) and depression (26.28±8.29 vs. 23.58±7.67, t_(621)_=-3.54, p<0.001) was significantly associated with greater perceived stress (see Table 5).

### Predictors of medical school-related stress

Significant socio-demographic and clinical correlates of medical school stress were entered into a multiple regression model using the forced entry method. Reported funding of medical school as difficult (B=2.881, p<0.001), parents’ choice as a motivation for a career in medicine (B=-3.105, p<0.001), poor adherence to faith (B=2.376, p<0.001), problem drinking (B=5.196, p<0.001), and anxiety (B=2.231, p<0.002) were predictors of perceived stress encountered in medical school (Table 6).

## Discussion

In this study, we evaluated the relationship between perceived stress among medical students, their use of alcohol and other psychoactive substances, and psychiatric morbidity (anxiety symptoms and depressive symptoms). Overall medical students reported high rates of perceived stress, with a sizable proportion reporting alcohol use. Anxiety and depression symptoms were common. There was a significant association between greater perceived stress and poor funding, parental preference influencing the choice of study, self-rated poor adherence to religious faith and anxiety symptoms.

**Table 4:** Gender differences on the perceived stress in medical students

PMSS	Total		Females (n=264)		Males (n=359)		*t*	p-value	95% CI
	Mean	SD	Mean	SD	Mean	SD			
Feelings of anonymity and isolation	1.90	1.16	1.88	1.17	1.91	1.16	-0.31	0.76	-0.22 – 0.16
Inability to endure long hours	1.28	1.15	1.27	1.09	1.28	1.20	-0.12	0.90	-0.20 - 0.17
Unclear expectations of faculty	1.14	1.01	1.09	0.91	1.18	1.08	-1.03	0.30	-0.25 – 0.08
Medical training controls whole life	2.27	1.28	2.20	1.31	2.32	1.25	-1.22	0.22	-0.33 -0.08
Unable to master entire knowledge	1.80	1.26	1.81	1.19	1.79	1.31	0.19	0.85	-0.18 -0.22
Demanding physician role	2.07	1.14	2.03	1.15	2.09	1.13	-0.67	0.51	-0.24 -0.12
Competitive medical school	2.40	1.16	2.56	1.14	2.28	1.17	2.95	0.003	0.09 -0.46
Education as baptism of fire	2.02	1.19	1.95	1.19	2.08	1.19	-1.25	0.21	-0.31 – 0.07
Success despite administration	2.01	1.05	1.98	1.06	2.04	1.05	-0.76	0.45	-0.23 – 0.10
Medical school cold, impersonal	1.85	1.10	1.77	1.08	1.92	1.10	-1.66	0.10	-0.32 – 0.03
Medical school more threat than challenge	1.45	1.21	1.30	1.15	1.56	1.25	-2.70	0.01	-0.46 - -0.07
Worries about personal finances	2.18	1.28	1.90	1.29	2.39	1.24	-4.80	0.001	-0.69 - -0.29
Worries about accommodation	1.79	1.34	1.67	1.33	1.88	1.34	-1.87	0.06	-0.42- 0.01
Total	24.15	7.87	23.46	7.44	24.66	8.15	-1.89	0.06	-2.46 – 0.05

Overall, female students reported lower rates of perceived stress compared to their male counterparts, a finding inconsistent with a similar study from Pakistan.^4^ Shah and colleagues note that cultural restrictions placed on women in Pakistan limit involvement in a socio-cultural activity that may reduce stress. In contrast, though restrictions on what women may do in society exist in Nigeria, they are probably more specific to those in the lower social class. A sizable proportion of females who undergo a degree in medicine come from middle to upper-class families.

Male students were more concerned about personal finances, which contrasts with reports from Europe where female students were more likely to complain about finances.^30^^,^^31^ It has been theorised that females in Europe and North America worry more about finances because males would prefer to drop out of school to work, unlike females who fear that dropping out of school and working would earn them less starting pay compared to males. The exact reason for the discrepancy in this environment is unknown. We hypothesize however that, male medical students would probably be less likely to get funds from parents/relatives, or may spend more.

Students who rated their ease of obtaining funds for medical school as difficult were significantly more likely to report stress even after adjusting for other variables. Most students get funding for medical school from family/relatives. Student loans are non-existent in Nigeria, and the few who are on scholarships are under pressure to maintain satisfactory performance or risk losing their scholarship. In the face of dwindling subventions from the government to universities, higher institutions routinely increase fees to remain open. This way, they pass on the cost of running the school to the students. For private medical schools, the cost of tuition is quite prohibitive. This study provides evidence that students who are unable to fund their courses have higher associated perceived stress. Stress has been consistently reported to impact academic performance.^32^

**Table 5:** Correlates of perceived stress

Variable		PMSS Mean (SD)	t/F	p-value
Age				
	≤24 years	23.79 (7.35)	-1.13	0.26
	>24 years	24.51 (8.35)		
Gender				
	Female	23.46 (7.44)	1.89	0.06
	Male	24.66 (8.15)		
Previous degree				
	Yes	22.36 (6.63)	-1.98	0.05
	No	24.37 (7.99)		
Source of funding				
	Self-funded	23.57 (8.55)	0.22	0.80
	Parents/Family	24.16 (7.76)		
	Scholarship	24.76 (8.82)		
Adherence to faith				
	Strong	22.31(7.47)	39.44	0.001
	Fair	26.34 (7.53)		
	Poor	30.60 (6.73)		
Ease of funding course				
	Easy	22.02 (8.77)	-3.37	0.001
	Difficult	24.68 (7.55)		
Motivation to study medicine?				
	Parents decision	27.20 (7.98)	10.40	0.001
	Role model	24.84 (7.88)		
	Financial stability	27.22 (8.81)		
	Desire to help others	22.96 (7.38)		
Alcohol use (AUDIT)				
	No problem	23.63 (7.63)	-5.53	0.001
	Problem drinking	29.74 (8.37)		
Other psychoactive substance use (DAST-10)				
	Minimal risk	23.99 (7.76)	-1.90	0.06
	Moderate-Severe risk	26.38 (9.18)		
Anxiety (HADS-A)				
	Absent	23.20 (7.65)	-4.87	0.001
	Present	26.54 (7.95)		
Depression (HADS-D)				
	Absent	23.58 (7.67)	-3.54	0.001
	Present	26.28 (8.29)		

Students whose level of adherence to their faith was good were less likely to report perceived medical school-related stress. Though the concept of spirituality and religiosity are divergent, some argue that there may be a link. In New Zealand, a study of the impact of religiosity on coping among medical students showed that religious affiliation correlated well with better coping skills. In this same study, students with greater religiosity were better able to handle stress based on the confession of faith, hope, and love.^33^ Furthermore, a survey of medical students in Trinidad and Tobago showed that those who regarded their faith as important and actively practiced it were less likely to report depressive symptoms and to report burnout.^1^

**Table 6:** Predictors of perceived medical school stress

Variable	B	Standard deviation	*t*	p-value
Did not have a previous higher degree	1.705	0.959	1.78	0.076
Funding for medical school not easy	2.881	0.748	3.85	0.001
Parent choice as motivation for course	-3.105	0.621	-5.00	0.001
Poor adherence to faith	2.376	0.308	7.725	0.001
Problem drinking	5.196	1.077	4.82	0.001
Anxiety	2.231	0.709	3.15	0.002
Depression	0.648	0.786	0.825	0.410

Although most students did not report alcohol use problems, those who reported problem drinking had significantly more medical school-related stress. The prevalence reported from this study is lower than the rate of 13.6% from Ilorin, North Central Nigeria.^34^ Under-reporting may be responsible for this lower rate. In a recent review of substance use patterns among medical students, psychoactive substance use was low among medical students compared to other university undergraduates. The commonest substance used was alcohol, followed by tobacco and cannabis,^35^ Problem drinking was also predictive of greater severity of stress. Alcohol abuse is a common maladaptive coping mechanism used by university students in Nigeria,^34^ and psychological distress has been shown to be associated with problem drinking among undergraduates in Africa.^36^^-^^38^ Alcohol consumption could also be a means to relieve symptoms of anxiety and/or depression, which was associated with higher levels of stress in this study. However, the cross-sectional design of this study limits the ascertainment of causality between alcohol abuse and psychiatric morbidity.

Over a quarter of the participants in this study report significant anxiety symptoms, and about a quarter had significant depressive symptoms. The prevalence of depression and anxiety symptoms are consistent with previous studies on psychiatric morbidity globally among medical students.^39^^,^^40^ It is not possible to determine causality or to ascertain the direction of the relationship between stress, psychoactive substance use, and psychiatric morbidity. However, the prevalence observed among a representative sample of medical students from Nigeria suggests that medical educators should be mindful of the effect of poor mental health on academic excellence in medical schools. Poor mental health indices may be attributed to the high expectations from parents, relatives and teachers and the high level of competition among students in medical schools. Recently, the agency that regulates undergraduate medical education in Nigeria has set out to ensure that medical schools adhere to their allotted quota when training students. As a result, schools which admitted more than their allotted quota need to reduce the number of its students or risk losing its accreditation. This has led to greater competition for placement in senior classes among students. ^41^ Students on scholarships could also have fears about losing scholarships if they are unable to maintain high grades consistently. The positive association between anxiety and depressive symptoms with perceived stress is consistent with findings of increased psychiatric morbidity linked to stress among medical students.^42^^,^^43^  Unfortunately, over the years the duration of training in mental health within the syllabus of medical schools in Nigeria is becoming increasingly shorter. Knowledge of psychological reactions to stress and recognition and management of resulting psychiatric morbidity would ‘demystify’ and reduce the stigma surrounding these conditions, enabling affected students to apply more adaptive coping skills and more readily seek help from appropriate sources when overwhelmed.

Our findings should be interpreted with the following limitations in mind. First, the cross-sectional design of this study did not enable us to ascertain the temporal association between perceived stress and its correlates. Second, an objective assessment of the academic performance of the study participants was not done, due to logistic constraints the authors viewed would arise from getting medical schools to release performance evaluation of participants. We also feared that participation rates in the study would be poor if students knew we would ask for their performance evaluation from their school. Third, other possible correlates like, occupation of parents, were not ascertained. In spite of this, the representativeness and large sample size in this study provides a valuable improvement on available evidence. We also suggest that future studies comparing stress across student disciplines and the general population be conducted to identify if perceived high-stress levels are related to the course of study.

## Recommendations

The findings from this study are a call to educators in medical schools in Nigeria in particular and those in sub-Saharan Africa in general to improve mental health awareness in their curriculum, and foster mental health promotion programmes (such as education about risks of alcohol abuse, and adaptive ways to cope with stress) among undergraduate medical students. This would go a long way to mitigate the impact of psychiatric morbidity, reduce stigma associated with psychological problems and facilitate early help-seeking for students who develop mental health problems. Furthermore, to reduce the gap between available personnel and medical manpower needs in developing countries, governments should subsidise medical education, either through loans to students or bearing a large portion of education costs. Given the significant concerns of the medical students who participated in this study about their finances and its deleterious effect on mental health and stress. We also recommend that future research with a prospective design be undertaken to establish the temporal relationship between perceived stress and relevant socio-demographic and clinical correlates.

## Conflict of Interest

The authors declare that they have no conflict of interest.
